# The roles and regulation of the actin cytoskeleton, intermediate filaments and microtubules in smooth muscle cell migration

**DOI:** 10.1186/s12931-017-0544-7

**Published:** 2017-04-08

**Authors:** Dale D. Tang, Brennan D. Gerlach

**Affiliations:** Department of Molecular and Cellular Physiology, Albany Medical College, 47 New Scotland Avenue, MC-8, Albany, NY 12208 USA

## Abstract

Smooth muscle cell migration has been implicated in the development of respiratory and cardiovascular systems; and airway/vascular remodeling. Cell migration is a polarized cellular process involving a protrusive cell front and a retracting trailing rear. There are three cytoskeletal systems in mammalian cells: the actin cytoskeleton, the intermediate filament network, and microtubules; all of which regulate all or part of the migrated process. The dynamic actin cytoskeleton spatially and temporally regulates protrusion, adhesions, contraction, and retraction from the cell front to the rear. c-Abl tyrosine kinase plays a critical role in regulating actin dynamics and migration of airway smooth muscle cells and nonmuscle cells. Recent studies suggest that intermediate filaments undergo reorganization during migration, which coordinates focal adhesion dynamics, cell contraction, and nucleus rigidity. In particular, vimentin intermediate filaments undergo phosphorylation and reorientation in smooth muscle cells, which may regulate cell contraction and focal adhesion assembly/disassembly. Motile cells are characterized by a front-rear polarization of the microtubule framework, which regulates all essential processes leading to cell migration through its role in cell mechanics, intracellular trafficking, and signaling. This review recapitulates our current knowledge how the three cytoskeletal systems spatially and temporally modulate the migratory properties of cells. We also summarize the potential role of migration-associated biomolecules in lung and vascular diseases.

## Background

Smooth muscle cell migration plays an essential role in tube formation of hollow organs such as the airways and blood vessels during development. Smooth muscle cell motility has also been implicated in the pathogenesis of airway remodeling, a key feature of asthma. In addition to hyperplasia and hypertrophy, airway smooth muscle cell migration contributes to the development of airway remodeling. Smooth muscle thickening in the airways may stem from migration of proliferating cells in the muscle bundles or recruitment of circulating precursor cells to the smooth muscle layer [1–3].

**Fig. 1:**
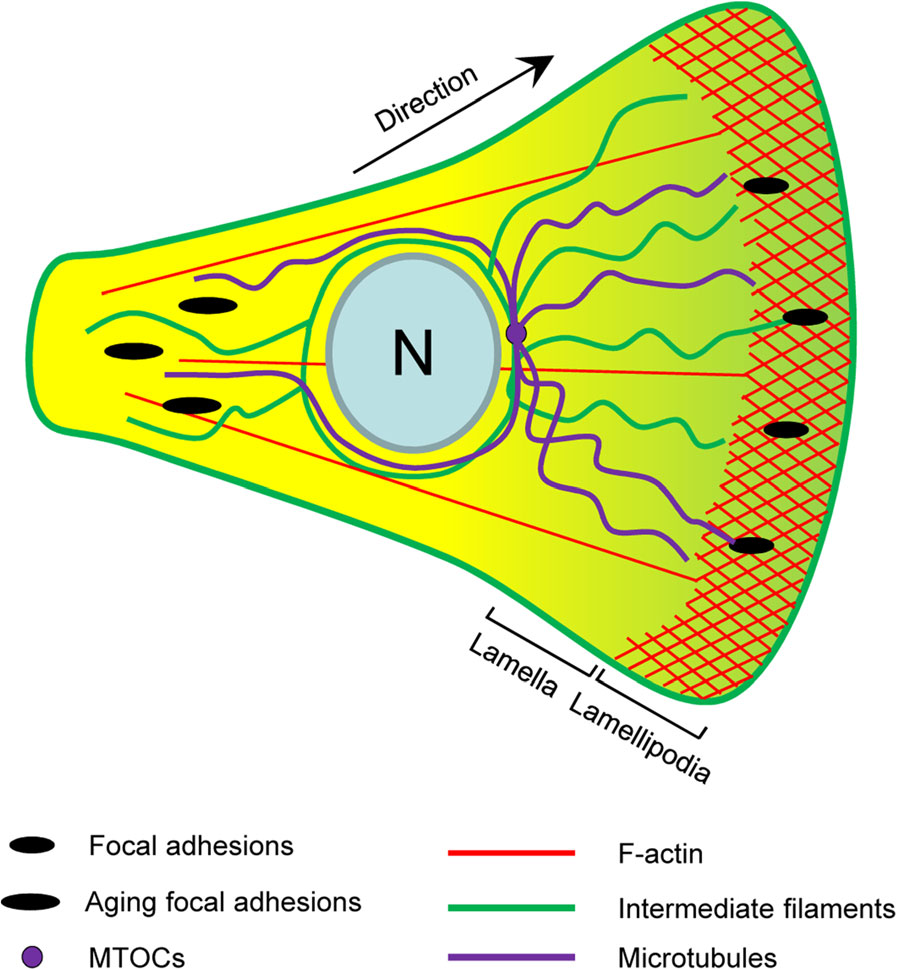
A. Schematic illustration of major cytoskeletal components in motile cells. Lamellipodia and focal adhesions are located in the front of motile cells. The cross-hatched region represents the actin framework in lamellipodia. F-actin is present throughout the cell body, which interacts with myosin to generate traction force. Aging focal adhesions in the rear are disassembled to allow for cell retraction. Intermediate filaments surround the nucleus (N), some of which associate with focal adhesions in lamellipodia. Intermediate filaments modulate focal adhesion dynamics and cell contraction. Microtubules are polarized along the direction of migration and accumulate toward the front of the cell. Microtubule organizing centers (MOTCs) are localized in the front of the nucleus. Through their roles in mechanics, trafficking and signaling, polarized microtubules facilitate all important events leading to cell migration

In general, cell migration includes the cycles of the following four steps. First, in response to guidance cues and adhesive proteins in the extracellular matrix (ECM), cells form a protrusion called lamellipodia at the front. Second, new focal adhesions are formed in the front of motile cells to strengthen their attachment to the ECM. Third, actomyosin activity increases to induce retraction of the rear. Fourth, focal adhesions at the cell rear are disassembled to allow whole cell body to move forward [1, 3, 4]. There is a wealth of evidence to suggest that the actin cytoskeleton, the intermediate filament network, and microtubules are involved in the regulation of cell motility (Fig. 1). This review will summarize our current understanding of physiological properties of the three cytoskeletal systems in cell migration in general and in smooth muscle cell migration in particular. The potential role of cell migration regulators in lung and vascular diseases is also reviewed.

## Roles of dynamic actin cytoskeleton and actin-associated proteins in cell migration

The actin cytoskeleton undergoes dynamic assembly and disassembly during cell crawling, which regulates protrusion formation, focal adhesion assembly/disassembly, and contractile filament organization. The disruption of the actin cytoskeleton inhibits cell migration and adhesion [1, 3, 4]. The dynamic actin architecture is regulated by a variety of actin-associated protein and signaling pathways.

### Lamellipodial formation is driven by local actin dynamics, and regulated by actin-associated proteins

The lamellipodia are thin, sheet-like membrane protrusions of motile cells. During migration, cells extend the membrane forward to explore their environment. If the front surrounding is suitable, cells will move forward. Otherwise, cells will retract to avoid inadequate environment. However, the extent of protrusion at the front is greater than retraction. Thus, the cyclic extension and retraction of the lamellipodium facilitate cell movement forward [1, 3–5]. The dynamic formation of lamellipodia is regulated by local actin filament assembly and disassembly.

**Fig. 2:**
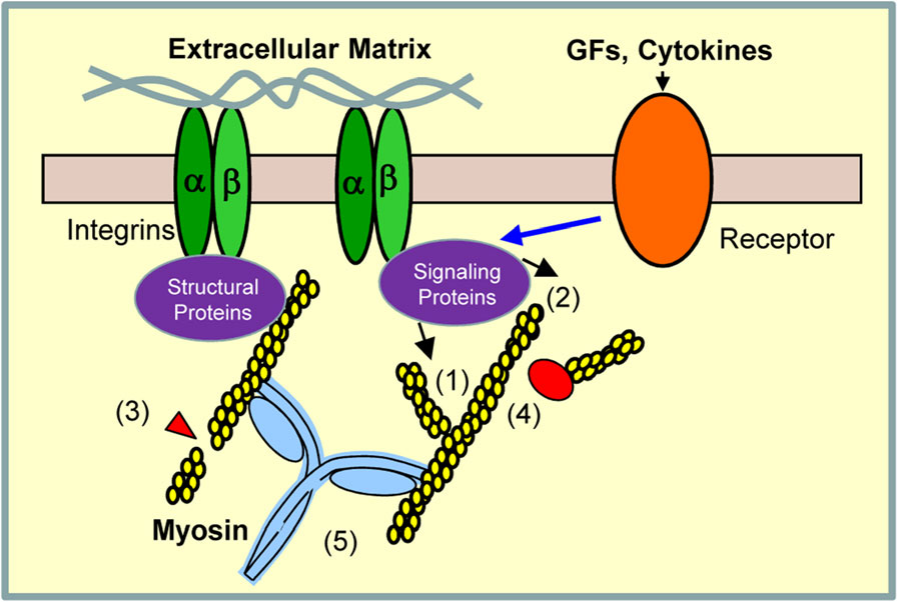
Focal adhesion formation, actin dynamics and actomyosin activity in motile cells. Engagement of integrins with the extracellular matrix recruits structural proteins (talin, vinculin, ILK, PINCH, parvins, α-actinin, etc.) and signaling proteins (Cdc42, c-Abl, cortactin, FAK, paxillin, Abi1, etc.) to the near integrin region, which promotes focal adhesion formation (see detailed molecular interactions at focal adhesions in reference [2]). Signaling proteins activate N-WASP and the Arp2/3 complex, which induce actin filament branching (1). Activation of profilin-1, VASP and mDia promotes actin filament elongation (2). Activation of gelsolin and cofilin results in actin filament severing and depolymerization (3). GMF-γ promotes actin filament debranching (4). Myosin light chain phosphorylation triggers actomyosin activity and leads to cell contraction (5). Soluble cues activate receptors (e.g. growth factor receptors, cytokine receptors) and signaling proteins, which promote actin filament polymerization and focal adhesion assembly (See details in text). GFs, growth factors

There are two patterns of actin filament assembly in the lamellipodia, branching and elongation, which promote the formation of the actin “mesh” in the cell protrusion. Furthermore, actin depolymerization and debranching transpires during migration, facilitating the dynamic remodeling of the actin network, and the cyclic extension and retraction of lamellipodia (Fig. 2).

**Fig. 3:**
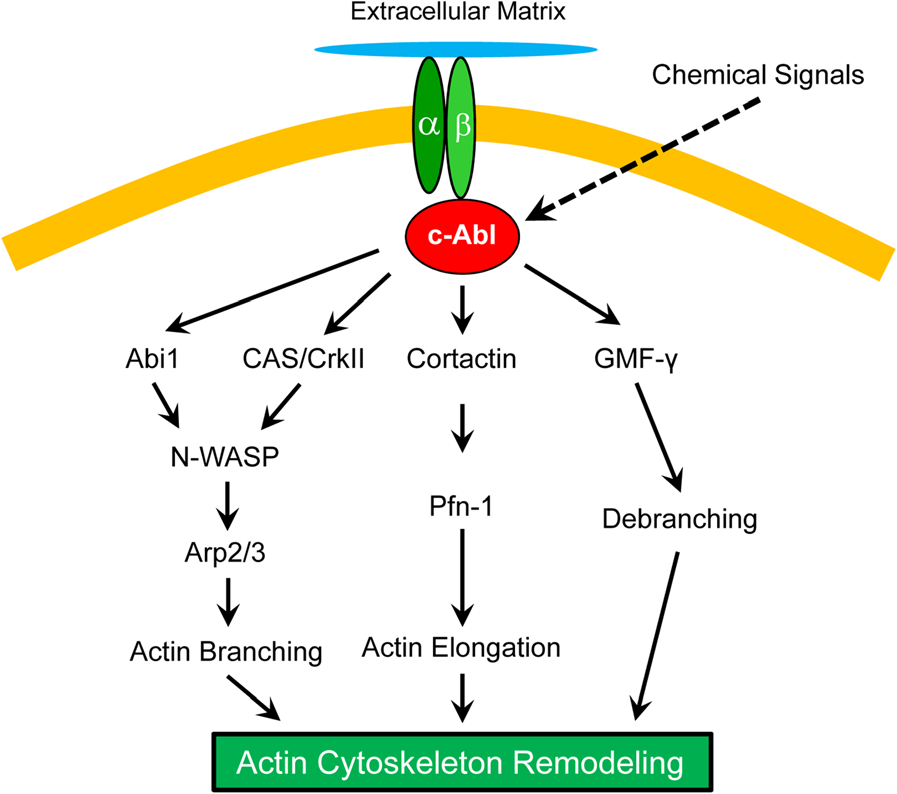
Regulation of airway smooth muscle cell migration by c-Abl tyrosine kinase. c-Abl is recruited to the leading edge by integrin β_1_, which activates the downstream pathways and regulates actin cytoskeletal remodeling in lamellipodia. Abi1, Abl interactor 1; Arp2/3, Actin-related protein 2/3; CAS, CrkII-associated substrate; GMF-γ, glia maturation factor-γ; N-WASP, neuronal Wiskott-Aldrich Syndrome Protein; Pfn-1, profilin-1

Actin filament branching is largely mediated by the Arp2/3 complex, which can attach to a mother filament, and induce daughter filament growth at 70° angle of mother filaments [4, 6]. The activity of the Arp2/3 complex is controlled by nucleation promoting factors such as neuronal Wiskott-Aldrich Syndrome Protein (N-WASP) and WASP-family verprolin-homologous protein (WAVE), which are in turn modulated by upstream regulators. Upon growth factor receptor ligation and cell adhesion, the small GTPases Cdc42 and Rac1 are able to bind to the GTP-binding domain of N-WASP/WAVE, activating N-WASP/WAVE and promoting the Arp2/3 complex-mediated actin filament branching [4, 6–8]. Recent studies suggest that the pleckstrin homology and RhoGEF domain containing G3 (PLEKHG3) protein is a GEF for both Rac1 and Cdc42. PLEKHG3 is recruited and selectively binds to new F-actin at the leading edge of migrating fibroblasts. Moreover, PLEKHG3 is regulated by phosphatidylinositol-4,5-bisphosphate 3-kinase (PI3K). However, it is currently unknown which PI3K isoforms are responsible for PLEKHG3 activation [9].

c-Abl is a non-receptor protein tyrosine kinase that plays an important role in regulating smooth muscle contraction, cell proliferation, and cytokinesis [2, 10–17]. Our recent results suggest that c-Abl is also critical for the regulation of smooth muscle cell migration [2, 3]. c-Abl is necessary for human airway smooth muscle cell migration. During airway smooth muscle cell migration, c-Abl is recruited to the leading edge and activated by integrin β_1_ [3]. c-Abl regulates the phosphorylation of the actin-regulatory protein cortactin, which may control the activation of N-WASP, and promote actin filament remodeling in lamellipodia [2, 3, 18]. Furthermore, the adapter protein Abi1 is capable of stimulating N-WASP in smooth muscle upon external activation [2, 13]. Abi1 is necessary for cell contraction and the movement of a variety of cell types including smooth muscle cells [13, 19] (our unpublished data). Abi1 activation in smooth muscle is also regulated by c-Abl tyrosine kinase [2, 13]. Moreover, external cues activate c-Abl tyrosine kinase in smooth muscle, which regulates phosphorylation of Crk-associated substrate (CAS), the coupling of CAS with CrkII and N-WASP activation [3, 6, 11, 20, 21] (Fig. 3). The role of c-Abl in modulating cortactin, Abi1 and CAS has been supported by studies on other cell types including fibroblasts and epithelial cells [18, 22, 23].

Elongation of actin filaments in smooth muscle is mediated by a number of proteins. Profilin-1 (Pfn-1) is recruited to the leading edge of motile smooth muscle cells, which promotes the transport of actin monomers to the barbed end of actin filaments in the cell protrusion [3]. The recruitment of Pfn-1 to the cell leading edge is regulated by the c-Abl-cortactin pathway [2, 3]. Moreover, vasodilator-stimulated phosphoprotein (VASP) undergoes phosphorylation at Ser-239 during cell adhesion and invasion, which promotes filament polymerization and smooth muscle cell motility [24, 25]. Additionally, the Rho effector formins (mDia) can nucleate and polymerize actin filaments at barbed end and enhance cell migration [1].

In addition, actin dynamics is able to promote the recruitment of β-catenin to N-cadherin in smooth muscle, which strengthens the cadherin-mediated intercellular linkage [2, 26]. Cadherin-mediated cell-cell adhesion facilitates collective cell migration and development [27].

Since cells have limited actin monomers, rapid polymerization of existing actin filaments cannot be persistent without being balanced by rapid depolymerization. The capping protein CapZ binds to the barbed end, preventing actin filament extension. The actin severing protein gelsolin interacts with aging filaments and severs them to short actin filaments. The actin-depolymerization protein ADF/coflin has higher affinity for ADP-actin fragments, which eventually leads to actin filament depolymerization [8] (Fig. 2).

Disassembly of the actin “mesh” is also facilitated by debranching. Glia maturation factor-γ (GMF-γ) is a 17 kDa member of the ADF/cofilin family that is capable of inducing actin depolymerization and debranching [28]. GMF-γ is able to bind to the Arp2/3 complex at the junction of mother filaments and daughter filaments, which induces debranching of the actin meshwork [28, 29]. Subsequently, debranched filaments are severed by gelsolin and depolymerized by ADF/cofilin, which eventually generate more G-actin pools for subsequent rounds of actin filament polymerization and branching [4, 28, 29]. Knockdown of GMF-γ attenuates the migration of neutrophils and T-lymphocytes [30, 31]. Our recent studies show that GMF-γ is necessary for human airway smooth muscle cell movement [32]. Contractile stimulation induces c-Abl-dependent GMF-γ phosphorylation at Tyr-104, which regulates airway smooth muscle cell contraction [2, 33]. Future studies are needed to assess whether GMF-γ phosphorylation at Tyr-104 by c-Abl has a role in regulating the migration and contraction of other cell types.

### Focal adhesions are actively assembled in the cell front and disassembled in the tail of motile cells

Focal adhesions are a type of adhesive contacts between the cell and the ECM. At focal adhesions, the extracellular domains of transmembrane integrins including α and β subunits connect with the ECM. The intracellular tails of integrins engages with linker proteins such as vinculin and talin, which in turn bind to the actin cytoskeleton (Fig. 2). Integrin-mediated focal adhesions link to the actin cytoskeleton, and allow cells to crawl on the ECM during migration. Nascent adhesions form at the leading edge and grow into focal complexes in lamellipodia. Some focal complexes undergo a rapid turnover at the rear of the lamellipodia whereas others become mature focal adhesions that will ultimately disassemble at the cell rear (Fig. 1).

During cell adhesion and migration, engagement of integrins with the specific motif of the ECM (e.g. RGD sequence) triggers focal adhesion assembly by inducing integrin aggregation and recruiting the structural proteins such as talin, vinculin, α-actinin, integrin-linked kinase (ILK), and parvins as well as signaling proteins including focal adhesion kinase (FAK), paxillin, Cdc42, and Rac1 [2]. The recruitment of structural proteins increases the sizes of focal complexes and strengthens the linkage of actin filaments to integrins in the lamellipodia. Signaling proteins in focal adhesions are able to initiate cascades to promote actin polymerization and other pathways. Adhesion-induced FAK activation mediates paxillin phosphorylation, which in turn activates N-WASP and actin filament polymerization in airway smooth muscle [2, 34–37] (Fig. 2). In addition, FAK is able to interact with Arp3 and enhances the Arp2/3-mediated actin polymerization and branching [38]. As described above, integrin-mediated c-Abl activation also promotes actin cytoskeletal remodeling near or within focal adhesions [2, 12]. Actin polymerization may conversely promote the recruitment of focal adhesion associated proteins to the plasma membrane [2, 39].

Focal adhesion formation in smooth muscle is also regulated by chemical stimulation. The activation of G-protein coupled receptor (GPCR) by agonists initiates the translocation of vinculin, α-actinin, ILK, and parvins to the integrin-associated sites on the plasma membrane [2]. In addition, activation of GPCR induces FAK tyrosine phosphorylation, paxillin phosphorylation and actin polymerization in smooth muscle [2, 40]. Moreover, the activation of GPCR and growth factor receptor stimulates c-Abl tyrosine kinase [2, 3, 15], which may regulate the functional state of CAS/CrkII and cortactin, and actin filament assembly in smooth muscle [2, 10, 13, 20, 34].

The mechanisms that control focal adhesion disassembly are under-investigated in smooth muscle cells. However, several studies from nonmuscle cells suggest that Prickle1 [41], Rap1-GTP-interacting adaptor molecule (RIAM) [42] and RhoJ [43] may promote focal contact disassembly. Prickle is a protein that is involved in convergent extension and cell migration. In gastric cancer MKN1 cells, Prickle1 accumulates at paxillin-associated focal contacts at the cell retraction site, and enhances microtubule-dependent focal adhesion disassembly [41]. RIAM may promote RhoA-dependent activation of the MEK-ERK1/2 pathway, which facilitates disassembly of focal adhesions in human melanoma cells [42]. RhoJ is a member of the Rho GTPase family that regulates cell motility, invasion, and focal adhesion numbers. In endothelial cells, active RhoJ interacts with the GIT-PIX complex, a regulator of focal adhesion disassembly, to enhance focal contact dissolution [43]. Moreover, microtubule dynamics are able to regulate focal adhesion disassembly (See *Microtubules and Cell Migration* in this review). Future studies are required to assess whether similar mechanisms exist in smooth muscle cells.

### Stress fiber formation and actomyosin activity are enhanced during cell migration

After nascent focal adhesions establish connection between the ECM and actin filaments, external signals induce stress fiber assembly and activate actomyosin ATPase, which generate traction force to propel the cell forward. Stress fibers are contractile bundles containing actin filaments and myosin II filaments. The engagement of integrins with the ECM activates the small GTPase RhoA, which is able to promote actin nucleation and stress fiber assembly by activating mDia. In addition, Rac1 activates p21-activated kinase (PAK), which phosphorylates and activates Lim kinase (LIMK). Activated LIMK mediates cofilin phosphorylation and inhibits actin filament depolymerization, thus limiting the amount of actin turnover and increasing stress fiber formation [39, 44].

RhoA can activate Rho kinase, which also promotes actomyosin ATPase activity by inhibiting myosin light chain phosphatase and/or by directly catalyzing myosin light chain phosphorylation [39, 44, 45]. RhoA activation may be mediated by RhoGEF Vav2 upon growth factor stimulation [46]. In addition, cell adhesion and the activation with growth factors are able to activate the tyrosine protein kinases Lck and Syk, which phosphorylates the RhoGEF Vav and activates RhoA [39, 47].

Myosin light chain phosphorylation may be also catalyzed by myosin light chain kinase during migration. Myosin light chain kinase is activated by Ca^2+^/calmodulin in myofibroblasts [48] and smooth muscle [1]. Furthermore, myosin light chain kinase is necessary for migration of various cell types including muscle cells [45, 48].

## Role of intermediate filaments in cell migration

Intermediate filaments (IFs) are widely distributed in the cytoplasm, providing mechanical and structural integrity for the cell [49, 50]. The genes encoding IF proteins are one of the largest family within the human genome, comprising over 65 genes encoding cytoskeletal and nucleoskeletal proteins that are each cell-type specific [49, 50]. The IF family is classified into 6 major types based upon sequence homology of the rod-like domain [50]. Type III IF proteins vimentin and desmin are major components of the IF networks in smooth muscle. The protein ratio of vimentin to desmin is 6:1 in airway smooth muscle [49–53].

There is evidence to suggest that cell movement is associated with vimentin protein expression. Cells with vimentin deficiency show slower migratory property [54, 55]. Knockdown of vimentin attenuates smooth muscle contraction [50, 52, 53], which may provide limited traction force to allow for migration [3, 8]. In contrast, higher vimentin expression promotes cell migration (e.g. during epithelial mesenchymal transition) [56]. Moreover, desmin has been implicated in smooth muscle contraction and cell migration [57, 58].

### Intermediate filaments regulate focal adhesion dynamics

**Fig. 4:**
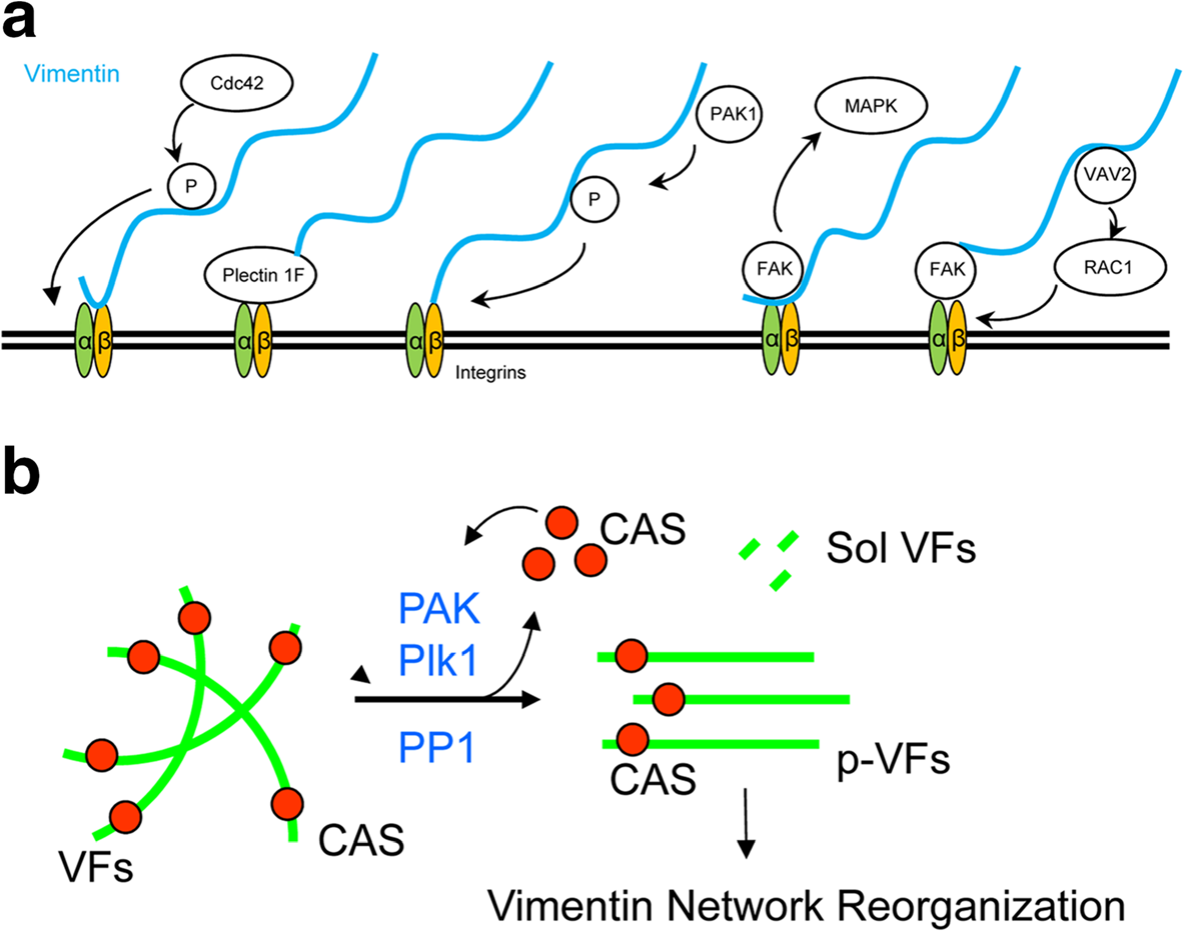
Vimentin intermediate filaments, focal adhesions and cell migration. **a** Vimentin filaments may directly bind to integrins β_1_ or β_3_, or indirectly to integrins via FAK or plectin 1F. Cdc42 or PAK induces vimentin phosphorylation, which activates integrins. Vimentin recruits VAV2 to focal adhesions to promote FAK activation. Interactions of vimentin filaments with focal adhesions can activate the MAPK pathway. **b** PAK1 and Plk1 are able to induce vimentin phosphorylation at Ser-56 in smooth muscle whereas protein phosphatase 1 (PP1) dephosphorylates vimentin. Vimentin phosphorylation induces vimentin disassembly and spatial reorientation, which regulates cell contraction and focal adhesion dynamics. Vimentin disassembly also releases CAS to affect actin dynamics. VFs, vimentin filaments; p-VFs, phospho-vimentin filaments; Sol-VFs, soluble vimentin filaments; CAS, Crk-associated substrate

Intermediate filaments have been shown to physically link to focal contacts in the protrusion of motile cells [56]. This raises the possibility that IFs may regulate focal adhesion dynamics directly, and thus cell migration. Higher vimentin expression in cells leads to the destabilization of desmosomes and increases focal adhesion dynamics to promote migration [56, 59]. Vimentin filaments underneath the plasma membrane interacts with the cytoplasmic tails of integrin β_3_ regulating the engagement of integrins with extracellular ligands and integrin clustering [60]. Vimentin filaments may also bind to integrin α_2_β_1_ directly or indirectly by FAK and plectin 1F [61, 62]. In addition, vimentin can recruit the Rac-GEF VAV2 to focal adhesions to promote FAK activation [56]. Moreover, uncoupling IFs from focal adhesions compromises the activation of FAK, Src and the downstream MAPK cascades to ERK1/2 and p38 [56, 63] (Fig. 4a). Thus, IFs may also regulate cell migration by controlling p38-mediated protrusion formation and stress fiber formation [1, 50].

The interaction of vimentin with the adhesive cell structure is modulated by vimentin phosphorylation. PAK1-mediated vimentin phosphorylation at Ser-56 leads to the spatial reorientation of vimentin filaments in smooth muscle cells, which may alter focal adhesion assembly [2, 54, 56, 64, 65]. PKCε-mediated phosphorylation of vimentin increases integrin translocation to the plasma membrane [56, 66], while Cdc2-mediated phosphorylation of vimentin induces integrin β_1_ activation [56].

### Intermediate filaments regulate cell contraction

As described earlier, cell contraction is critical for inducing retraction of the rear. In addition to focal contacts, vimentin intermediate filaments of smooth muscle attach to desmosomes on the plasma membrane and to dense bodies in the myoplasm. The dense bodies are also the locations to which contractile actin filaments attach. Thus, the physical linkage of vimentin filaments to dense bodies provides the structural base by which vimentin intermediate filaments may modulate smooth muscle cell contraction.

Vimentin intermediate filaments are required for smooth muscle contraction. Our previous studies have shown that vimentin knockdown by antisense oligonucleotides inhibits smooth muscle force development [50, 53]. Moreover, vimentin-deficient fibroblasts display impaired contractile capacity [55]. External stimulation induces vimentin phosphorylation at Ser-56, which leads to reorganization of the vimentin network, facilitating mechanical force transduction in smooth muscle. Vimentin phosphorylation at Ser-56 is catalyzed by p21-activated kinase 1 (PAK1) and polo-like kinase 1 (Plk1) in smooth muscle [50, 52, 67, 68]. Vimentin dephosphorylation at this residue is regulated protein phosphatase 1 in smooth muscle [49] (Fig. 4b). Vimentin phosphorylation at Ser-56 is also necessary to regulate the functions and/or locomotion of endothelial cells [69] and cancer cells [70].

### Intermediate filaments regulate nucleus rigidity

When cells move in a three-dimensional environment, the size of the nucleus influences the rate of migration. This is because the nucleus is the largest organelle inside the cell. Thus, alterations of nucleus rigidity affect the cell ability to squeeze in between matrix fibers. Lamins are the type IV intermediate filament proteins that are the major components of the nuclear membrane [50] and largely affect the mechanical property of the nucleus. Lamin A/C are overexpressed in prostate cancer tissues and knockdown of lamin A/C inhibits prostate cancer cell migration [71]. However, lamin A/C expression is reduced in gastric carcinoma, implying that lower lamin A/C expression may promote gastric cancer cell movement [72]. Therefore, the impact of nuclear lamins on nucleus rigidity and invasion is dependent upon cancer cell types and local environment. Since lamins are present in muscle cells [73], it is likely that nuclear lamins affect nucleus rigidity and modulate smooth muscle cell migration in tissues, a three-dimensional environment.

### Intermediate filaments interact with the actin cytoskeleton and microtubules

The vimentin network is able to regulate the actin cytoskeleton in several ways. First, vimentin phosphorylation at Ser-56 by PAK1 and Plk1 leads to its disassembly in smooth muscle, which results in the release of CAS from cytoskeletal vimentin. CAS translocates to the cell cortex and promotes the Arp2/3 complex-mediated actin polymerization and branching, and lamellipodial formation [2, 34, 35, 50, 52, 65, 67, 74] (Fig. 4b). Second, caldesmon is a component of microfilaments in all cells and thin filaments in smooth muscle cells. Caldesmon is able to interact with intermediate filaments and polymerized actin, and is required for maintaining the intermediate filament network and actin filaments in smooth muscle cells [75]. Caldesmon phosphorylation by the serine/threonine protein kinase PFTAIRE1 promotes its binding to F-actin and stress fiber formation in motile cells [76]. Third, CARMIL2 (capping protein, Arp2/3, myosin-I linker 2) is a molecule that regulates the activity of capping protein. During migration, dynamic vimentin filaments target CARMIL2 to the cell cortex, where CARMIL2 modulates capping protein activity and increases local actin filament assembly and protrusion formation [77].

As described earlier, increased expression of vimentin intermediate filaments enhances directed cell migration. Recent evidence suggests that the vimentin filament network assembles along the template of polarized microtubules. The longer-lived vimentin network then provides the template for future microtubule growth thus supporting and driving cell polarity and the directional persistence of migration [78]. This is further supported through previous micro-patterning studies showing that the vimentin filament network is crucial for microtubule organization, maintenance of cell polarity, and directional migration [79].

## Microtubules and cell migration

Microtubules are long and hollow cylinders made up of α- and β-tubulin dimers, which bind in a head-to-tail manner into protofilaments that associate laterally to form hollow tubes. Microtubule assembly is a polarized process that starts from one or several microtubule organizing centers (MTOCs). Typically, the centrosome serves as a major MTOC and stabilizes microtubule minus ends that are embedded in this complex structure. The plus ends of microtubules point towards the cell periphery. Although microtubule elongation transpires at both plus and minus ends, it is more rapid at plus ends [80]. Microtubule restructuring have been shown to regulate smooth muscle cell migration [81–84]. Through their roles in mechanics, trafficking and signaling, microtubules regulate lamellipodial formation and focal adhesion dynamics. Moreover, microtubules undergo polarization during migration, which regulates migration-associated events in a spatial and temporal manner.

### Microtubules facilitate protrusion formation

In motile cells, most microtubules do not enter lamellipodia; however, some microtubules, called pioneer microtubules, do extend to the protrusion sites. Because microtubules have ability to resist high compressive loads [85], it is likely that microtubule elongation in the protrusion may assist in pushing the membrane forward [80, 81]. Microtubule elongation is facilitated by several microtubule plus-end tracking proteins (+TIPs) such as end-binding protein (EB) EB1 and EB3, and several + TIP stabilizing factors such as adenomatous polyposis coli (APC) [80]. In addition, cytoplasmic linker associated proteins (CLAPs) may regulate microtubule assembly in the front of motile smooth muscle cells [81].

**Fig. 5:**
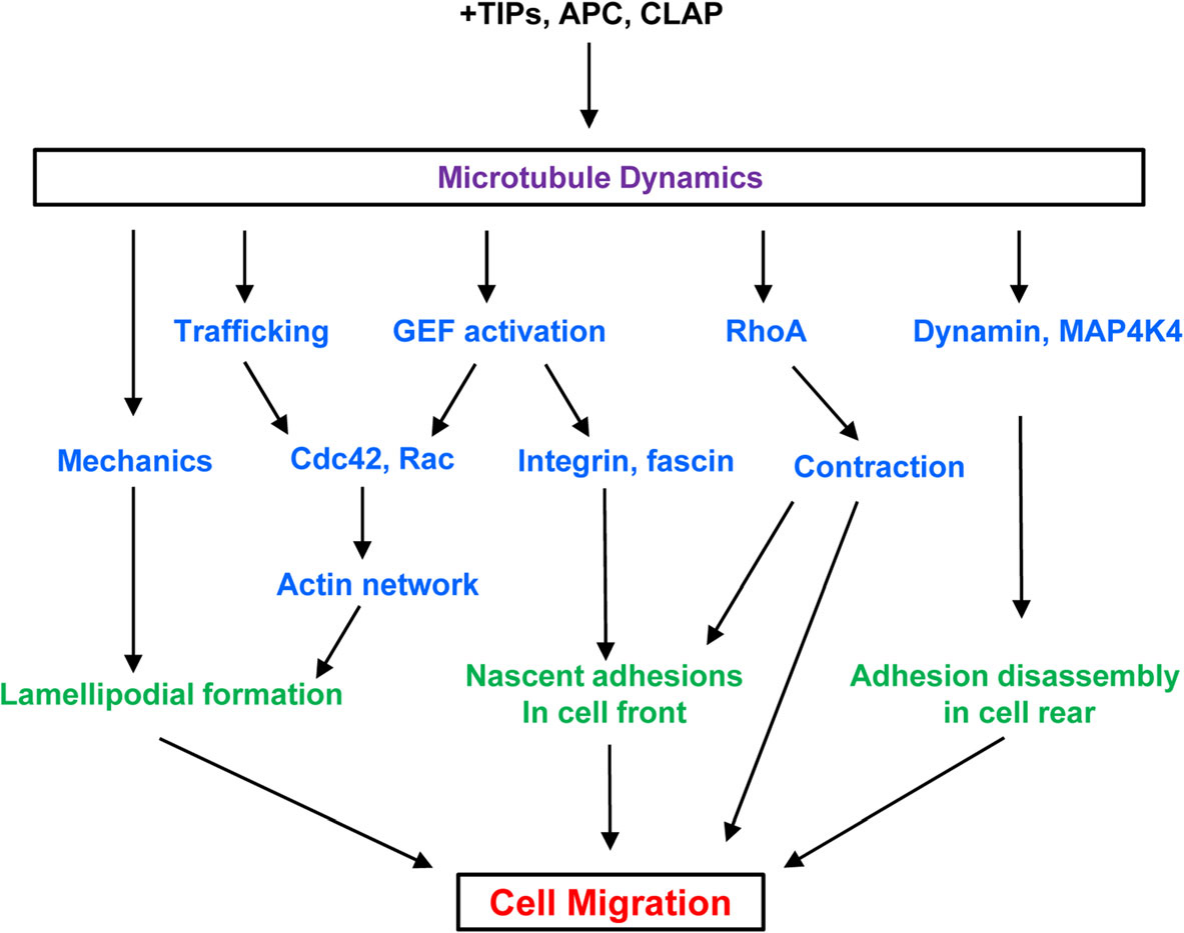
Cell migration regulated by microtubule-associated processes. Microtubule dynamics is regulated by plus-end trafficking proteins (+TIPs),+TIP stabilizing protein (e.g. adenomatous polyposis coli, APC), and cytoplasmic linker associated proteins (CLAPs). Microtubules regulate cell migration through their roles in mechanics, trafficking and signaling. GEF, guanine nucleotide exchange factor; MAP4K4, mitogen-activated protein kinase kinase kinase kinase 4.

Microtubules may promote the delivery of membrane vesicles that are essential for cell protrusion [86]. Microtubules can deliver recycling endosomes carrying membrane-associated signaling molecules (e.g. Rac, Cdc42, and the guanine nucleotide exchange factor βPIX) critical for cell migration [87, 88]. Moreover, microtubule assembly and disassembly are able to activate a growing number of GEFs to protrusion sites. GEFs activate the small GTPases that promote actin mesh reorganization and lamellipodial formation [80, 87, 88] (Fig. 5).

### Microtubules regulate focal adhesion dynamics

Microtubules are able to facilitate nascent focal complex assembly in the leading edge. Microtubule-dependent activation of the Rac-GEF TIAM2/STEF promotes the formation of new focal adhesions [89]. In addition, microtubules promote the polarized delivery of integrins to the leading-edge plasma membrane and participate in the growth of early focal adhesions [80, 90]. In recent years, microtubules are found to interact with fascin (an actin-binding and bundling protein), which contributes to fascin-dependent control of focal adhesion dynamics and cell migration speed [91]. Maturation of focal complexes in lamellipodia is facilitated by actomyosin-mediated contractile force. Microtubule depolymerization induces an increase in RhoA activity and cell contractility [92]. It is likely that changes in microtubule dynamics proximal to forming focal adhesions may locally increase cell contractility and, consequently, focal adhesion assembly [80]. RhoA-mediated contraction may also promote the retraction of the cell rear [80] (Fig. 5).

Microtubules have been shown to trigger the disassembly of mature focal adhesions in the cell rear. Treatment of cells with nocodazole results in the accumulation of integrins in mature focal adhesions, which is reversible after removal of nocodazole [80, 92]. Dynamic microtubules recurrently target mature focal adhesions, which disassemble at the cell rear, by interacting with plus end tracking proteins [93]. Dynamin localizes at focal contacts and is required for focal adhesion disassembly in migrating cells, probably by promoting internalization of integrin complexes [94]. Dynamin also interacts with microtubules, which suggests that microtubules could deliver dynamin to focal adhesions to trigger intergrin-associated endosome internalization [80, 95]. Moreover, quantitative proteomics suggest that mitogen-activated protein kinase kinase kinase kinase 4 (MAP4K4) is a focal adhesion regulator that associates with microtubules. Knockout of MAP4K4 stabilizes focal contacts and impairs cell migration. Microtubules may deliver MAP4K4 toward focal contacts through EB2 (ending binding 2), where MAP4K4 can, in turn, activate Arf6 via IQ motif and SEC7 domain-containing protein 1 (IQSEC1), a guanine nucleotide exchange factor specific for Arf6, and enhance focal adhesion dissolution [96].

### Microtubule network undergoes polarization during migration

Microtubules are able to regulate protrusion, focal adhesion assembly/disassembly and cell contraction locally. For directed migration to occur, microtubules are organized in a polarized manner to ensure spatial and temporal coordination of these events. In immobile cells, the microtubule framework is radially organized and shows no obvious polarization. In motile cells, the microtubule network is aligned with the axis of cell migration, which results from the orientation of the nucleus–centrosome axis parallel to the direction of migration, and from the organization of microtubules in an elongated and parallel array. In most cells, microtubules accumulate toward the front of the cell and MTOCs localize in the front of the nucleus towards the direction of migration [80, 93].

Polarization of the microtubule network facilitates trafficking of vesicles containing integrins and other molecules at the front to promote protrusion and focal contacts. Polarized microtubules may also assist mature focal adhesion disassembly in the rear by transporting molecules such as dynamin and MAP4K4 [80, 93, 96].

Chemical gradient of soluble or membrane-bound chemoattractant or the localized activation of integrins induces the recruitment and activation of the RhoGTPases Cdc42 and Rac, and PI3K-γ at the cell leading edge, which triggers microtubule polarization and directed cell migration. Cdc42 and PI3K-γ can activate glycogen synthase kinase 3 α/β (GSK3α/β), regulating APC, CLASPS and stabilizing microtubule-associated proteins (MAPs) at the plus end, which increases microtubule growth, capping, and stability. GSK3α/β also affects stabilizing MAPs, ACF7 (a microtubule-microfilament linker) and kinesin light chain along the microtubule network, which enhances the interaction of microtubules with actin and kinesin-mediated motility. The GSK3α/β-mediated microtubule dynamics is the key feature of microtubule polarization. In motile smooth muscle cells, receptor for hyaluronan-mediated motility (RHAMM) plays an important role in rear polarization of MTOCs and directed migration [80]. Spectrin-α (an actin-associated protein) colocalizes with RHAMM at the nodes of the actin net. Thus, spectrin-α interacts with RHAMM to regulate microtubule polarization in smooth muscle cells [82].

## Smooth muscle cell migration and diseases

It has been proposed that airway smooth muscle cell migration plays a role in the development of smooth muscle thickening in the asthmatic airways. Increases of the smooth muscle layer thickness in the asthmatic airways may be due to migration of smooth muscle cells in the muscle bundles [1–3]. In addition, there is evidence to suggest that vascular smooth muscle cell migration contributes to the progression of neointima formation after vascular injury [97]. Thus far, several biomolecules have been shown to regulate smooth muscle cell migration (at least in part) and the development of pulmonary and vascular diseases (Table 1). Some of them have been used as biotargets to develop new therapies to treat lung and vascular diseases.

**Table 1:** Role of migration‐associated biomolecules in lung and vascular diseases

Name	Role	Diseases	References
c‐Abl	Tyrosine kinase, promote migration	Asthma, PAH, atherosclerosis	[12, 98, 106, 107, 111]
β‐catenin	Junction protein, collective migration	Asthma	[104]
Cortactin	Adapter protein, promote protrusion	Asthma	[103]
FAK	Tyrosine kinase, adhesion, signaling	Vascular injury	[110]
Integrin β1	Mechanoreceptor, adhesion, signaling	Asthma, vascular injury	[102, 110]
mDia1	Actin‐associated protein, actin nucleation	Vascular Injury	[112]
Myosin	Contractile protein	Vascular injury	[97, 110]
p38MAPK	S/T kinase, promote locomotion	Asthma	[1, 99]
Pfn‐1	Actin‐associated protein, migration	Hypertension	[108]
PI3K‐γ	Kinase, regulate actin and microtubules	Asthma	[105]
RhoA/ROCK	S/T kinase, promote contraction	Asthma	[100, 101]
Vimentin	IF protein, promote contraction/motility	Vascular remodeling	[109]

*FAK* foca adhesion kinase, *IF* intermediate filament, *Pfn‐1* profilin‐1, *PAH* pulmonary arterial hypertension, *S/T* serine/threonine, *ROCK* Rho kinase

### Smooth muscle migration regulators and airway remodeling

#### c-Abl tyrosine kinase

As described earlier, c-Abl tyrosine kinase positively orchestrates airway smooth muscle migration by modulating actin network reorganization [3]. To assess its role in vivo, we have generated c-Abl smooth muscle conditional knockout mice. Allergen exposure leads to increases in the thickness of the airway smooth muscle layer in mice, which is reduced in c-Abl conditional knockout mice [12]. In addition, the expression of smooth muscle α-actin in the airways is upregulated in mice exposed to the allergen. But, c-Abl conditional knockout diminishes the upregulation of smooth muscle α-actin in the airways [12]. Furthermore, the role of c-Abl in airway smooth muscle thickening is supported by using the c-Abl inhibitor imatinib [98]. These results suggest that c-Abl mediated smooth muscle migration participates in the development of airway remodeling in the asthmatic animals.

#### p38MAPK

There is evidence that p38 inhibition reduced airway smooth muscle cell migration. Moreover, treatment with an inactive PAK1 attenuated p38 activation and airway smooth muscle migration [1]. Interestingly, inhibition of p38 suppressed airway remodeling in an animal model of asthma [99].

#### RhoA and Rho kinase

The roles of RhoA and Rho kinase in smooth muscle cell locomotion are well described [39, 44, 45]. Th2 cytokines could increase the expression of RhoA in airway smooth muscle [100]. Inhibition of the RhoA/Rho kinase hinders the development of airway remodeling in experimental asthma [101].

#### Others

β_1_ integrin is associated with asthma pathogenesis [102]. Treatment with RGD peptide blocks integrin activation and reduces airway remodeling in asthmatic animals [102]. A common cortactin gene variation has been found to confer susceptibility of severe asthma [103]. Since cortactin regulates smooth muscle cell protrusion formation [3], it is likely that cortactin-associated migration may contribute to asthma pathogenesis. β-catenin [104] and PI3K-γ [105] have been implicated in asthma pathogenesis. Despite their involvement of cell migration, we do not know exactly how these proteins contribute to airway remodeling.

### Vascular smooth muscle migration and vascular diseases

#### Vascular remodeling

Smooth muscle cells play a critical role in the pathogenesis of vascular diseases and its clinical manifestations. Chronic pulmonary arterial hypertension is characterized by vascular remodeling. c-Abl tyrosine kinase is involved in the pathogenesis of pulmonary arterial hypertension. Treatment of the c-Abl inhibitor imatinib relieves the symptoms of a patient with pulmonary arterial hypertension [106]. Results from Phase II and III clinical trials suggest that imatinib has potent and prolonged efficacy in patients with severe pulmonary arterial hypertension [107].

Vascular remodeling is also a key feature of systemic hypertension. Pfn-1 [108] and vimentin [109] have been shown to mediate vascular remodeling in animal models. Pfn-1 knockdown inhibits arterial remodeling in hypertensive rats whereas overexpression of Pfn-1 promotes vascular remodeling [108]. Flow-induced vascular remodeling may contribute to the development of hypertension. Flow-induced vascular remodeling is reduced in vimentin knockout mice [109].

#### Neointima formation

In addition to atherosclerosis, neointima formation is a major pathological process after percutaneous coronary intervention, bypass operation, or graft vasculopathy. It has been widely accepted that intimal smooth muscle cells in proliferative vascular diseases are derived largely from resident medial smooth muscle cells [97]. As mentioned earlier, β_1_ integrin and FAK are able to regulate cell migration by controlling dynamics of focal adhesions and the actin cytoskeleton [2, 34–38]. Myosin light chain phosphorylation modulates cell contraction to facilitate smooth muscle cell migration [39, 44]. Inhibition of β_1_ integrin expression, FAK phosphorylation and myosin activation is associated with reduced neointima formation in vivo [97, 110]. In addition, c-Abl has been implicated in the pathogenesis of atherosclerosis; inhibition of c-Abl by imatinib attenuates the progression of diabetes-associated atherosclerosis [111]. Furthermore, formin mDia1 has been shown to mediate neointima expansion in an animal model [112].

## Conclusions and perspectives

Elucidating the mechanisms of smooth muscle cell migration is a hot topic in smooth muscle biology and asthma research. The actin-associated proteins are able to regulate actin branching, elongation, debranching, depolymerization, focal adhesion dynamics, and contraction. c-Abl tyrosine kinase in smooth muscle plays a key role in modulating these cellular processes. Intermediate filaments coordinate focal adhesion assembly/disassembly, contraction, and nucleus rigidity. The vimentin intermediate filament network undergoes phosphorylation and spatial reorganization in smooth muscle, which regulates its function in smooth muscle. PAK1, Plk1 and PP1 are important molecules that regulate vimentin phosphorylation in smooth muscle. More studies are required to investigate the role and mechanisms of the intermediate filament network in smooth muscle cell migration. Although the role of microtubules in non-muscle cell motility has been described, their functions in smooth muscle cells remain to be elucidated. It will be interesting to develop animal models to verify the role of PAK1 and Plk1 in cell migration and airway/vascular remodeling in vivo. Furthermore, it will be very attractive to identify potential smooth muscle specific cell migration regulators that could be used to treat smooth muscle diseases such as asthma, hypertension, and vascular injury.

## Abbreviations

+TIPs: Microtubule plus-end tracking proteins
Abi1: Abl interactor 1
ADF: Actin-depolymerizing factor
APC: Adenomatous polyposis coli
c-Abl: Abelson tyrosine kinase
CARMIL2: Capping protein, Arp2/3, myosin-I linker 2
CAS: Crk-associated substrates
EB: End-binding protein
ECM: The extracellular matrix
ERK1/2: Extracellular signal-regulated kinase1/2
FAK: Focal adhesion kinase
GIT-PIX: G-protein-coupled receptor kinase-interacting target - Pak-interacting exchange factor
GMF-γ: Glia maturation factor-γ
GPCR: G-protein coupled receptor
GSK3α/β: Glycogen synthase kinase 3 α/β
ILK: Integrin-linked kinase
IQSEC1: IQ motif and SEC7 domain-containing protein 1
LIMK: Lim kinase
MAP4K4: Mitogen-activated protein kinase kinase kinase kinase 4
MAPK: Mitogen-activated protein kinase
MAPs: Microtubule-associated proteins
MTOCs: Microtubule organizing centers
N-WASP: Neuronal Wiskott-Aldrich Syndrome Protein
PAK1: p21-activated kinase 1
Pfn-1: Profilin-1
PI3K: Phosphatidylinositol-4,5-bisphosphate 3-kinase
PINCH: Particularly interesting new cysteine-histidine-rich protein
PLEKHG3: Pleckstrin homology and RhoGEF domain containing G3
Plk1: Polo-like kinase 1
PP1: Protein phosphatase 1
RHAMM: Receptor for hyaluronan-mediated motility
RIAM: Rap1-GTP-interacting adaptor molecule
Tiam2: T cell lymphoma invasion and metastasis 2
VASP: Vasodilator stimulated phosphoprotein
